# Supplementary figures and images for: Identification of key microRNAs and the underlying molecular mechanism in spinal cord ischemia-reperfusion injury in rats
Source: PeerJ. 2021 May 27;9:e11454. doi: 10.7717/peerj.11454 (PMC8164840; doi:10.7717/peerj.11454)

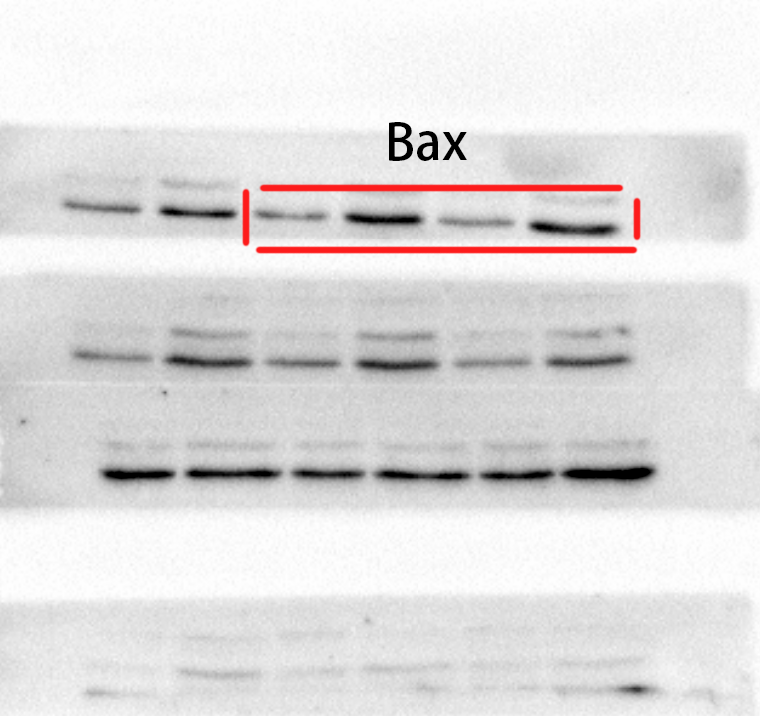

Supplement: Supplemental Information 2 [file peerj-09-11454-s002.zip › Uncropped GelsBlots-Western Blots/Uncropped GelsBlots of BAX.tif]

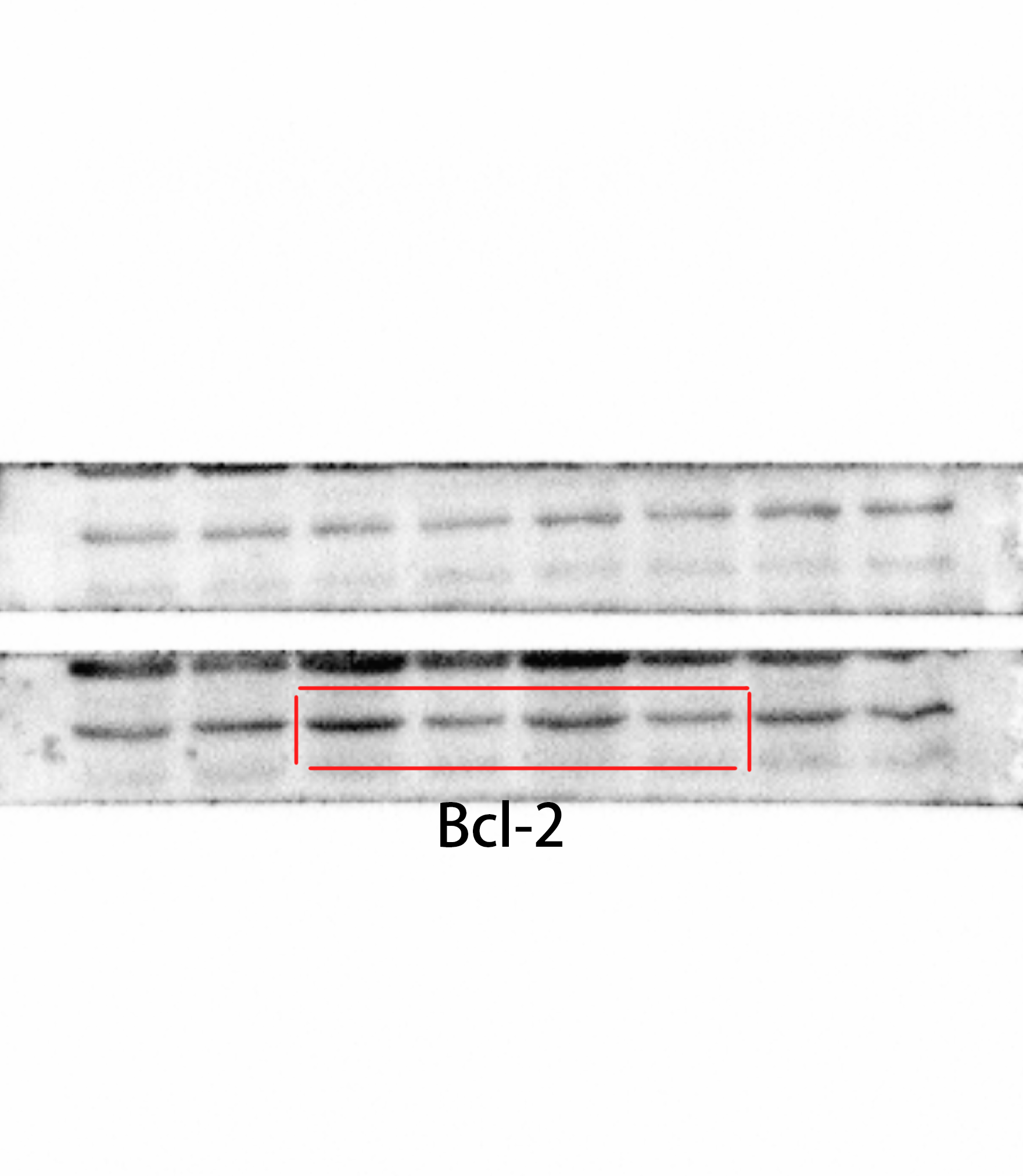

Supplement: Supplemental Information 2 [file peerj-09-11454-s002.zip › Uncropped GelsBlots-Western Blots/Uncropped GelsBlots of BCL-2.tif]

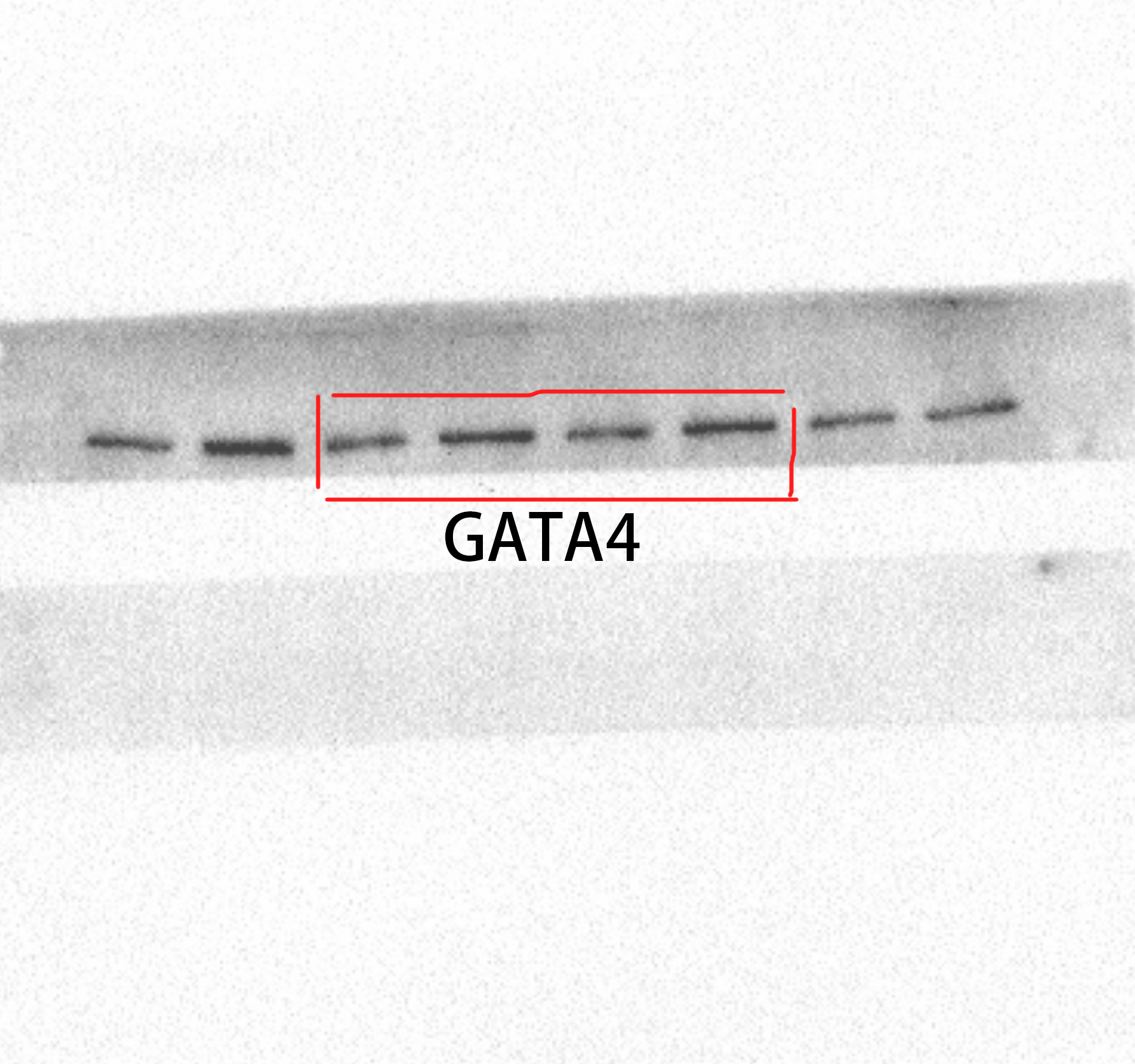

Supplement: Supplemental Information 2 [file peerj-09-11454-s002.zip › Uncropped GelsBlots-Western Blots/Uncropped GelsBlots of GATA4.tif]

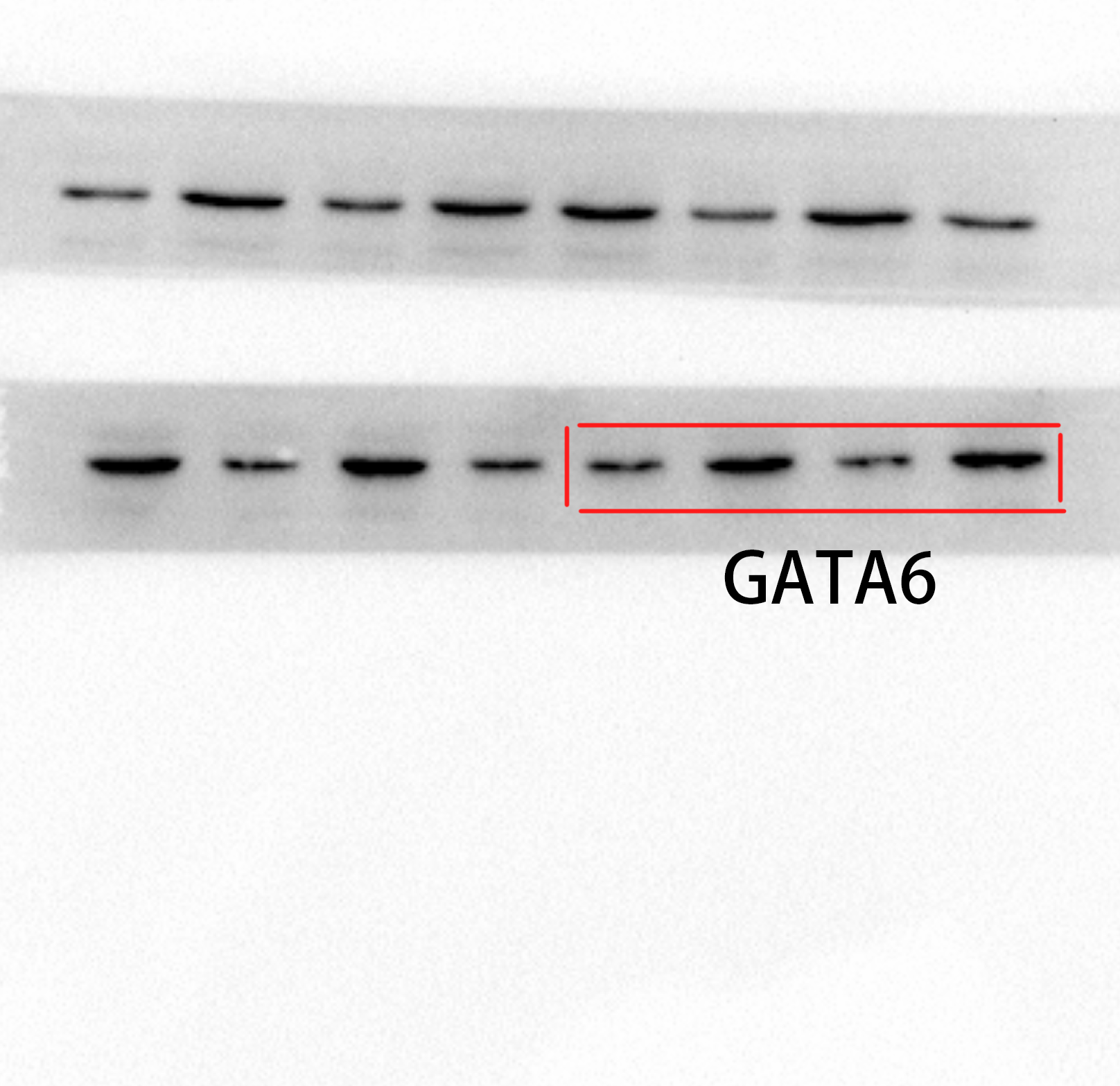

Supplement: Supplemental Information 2 [file peerj-09-11454-s002.zip › Uncropped GelsBlots-Western Blots/Uncropped GelsBlots of GATA6.tif]

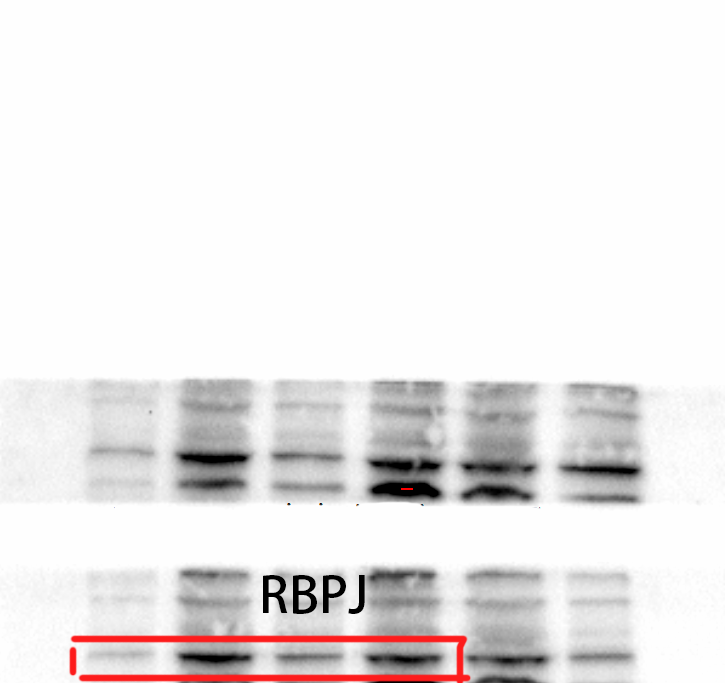

Supplement: Supplemental Information 2 [file peerj-09-11454-s002.zip › Uncropped GelsBlots-Western Blots/Uncropped GelsBlots of RBPJ.tif]

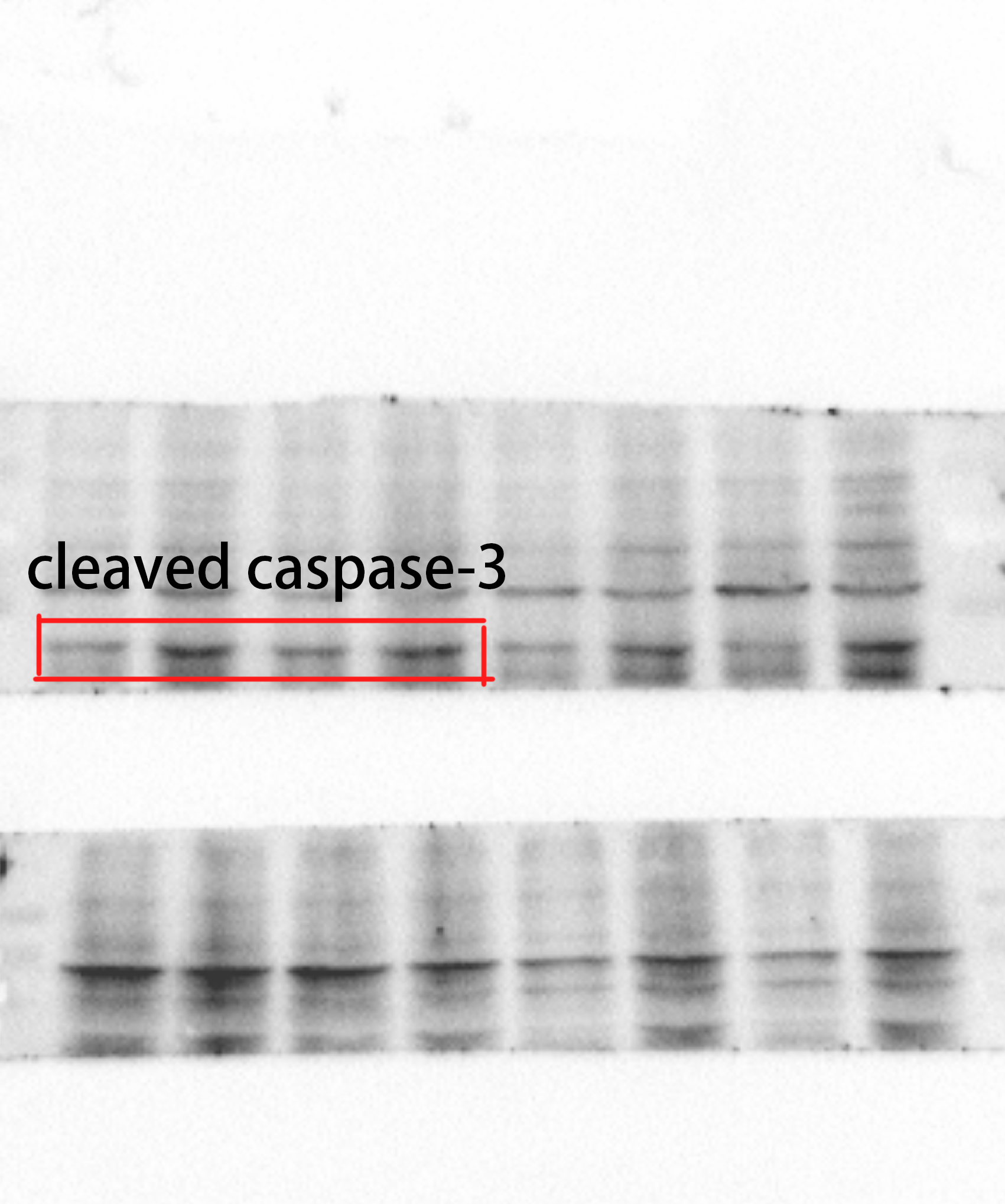

Supplement: Supplemental Information 2 [file peerj-09-11454-s002.zip › Uncropped GelsBlots-Western Blots/cleaved caspase-3.tif]
